# Brain Derived Neurotropic Factors in Speed vs. Inclined Treadmill in Young Adult Healthy Male With Occult Balance Disorder

**DOI:** 10.3389/fnint.2019.00033

**Published:** 2019-08-06

**Authors:** Stephanie T. Yulinda, Damayanti Tinduh, Lukitra Wardhani, Hening Laswati, Sony Wibisono, Melaniani Soenarnatalina

**Affiliations:** ^1^Department of Physical Medicine and Rehabilitation, Faculty of Medicine, Dr. Soetomo General Academic Hospital, Universitas Airlangga, Surabaya, Indonesia; ^2^Department of Internal Medicine, Faculty of Medicine, Dr. Soetomo General Academic Hospital, Universitas Airlangga, Surabaya, Indonesia; ^3^Department of Biostatistics and Population Studies, Faculty of Public Health, Universitas Airlangga, Surabaya, Indonesia

**Keywords:** brain derived neurotropic factor, occult balance disorder, speed treadmill, inclined treadmill, young adult healthy male

## Abstract

**Background**: There is an increase in fall risk among elders and young adults consecutively due to various causes. Occult balance disorder may be among the abnormal causes of falling in young adults as well as elders. The One Leg Stance (OLS) test is used to diagnose this balance performance; it’s a proven test to measure static balance function which would lead to dynamic balance function. It has been proven that aside from cardiopulmonary exercises, treadmill workout can be used as a dynamic balance exercise. The Brain Derived Neurotropic Factor (BDNF) increases balance function through the treadmill exercise (the inclination and speed). This hormone is one of the tropical hormones generated in neurons, muscles, hematopoietic tissue and it is characterized by neurons morphology regulation and neuroplasticity.

**Materials and Methods**: We divided 20 healthy young adult men to work out on inclination and speed groups treadmill exercise. The workout lasted for 2 weeks. We immediately observed the effect of exercise on serum BDNF as two tests were taken on before and 30 min after the workout.

**Result**: There were significant increases of pre-exercise serum BDNF level in speed group between the first and the last exercise (*p* = 0.001), post-exercise between the first day and the last exercise (*p* = 0.001). No significant increase of serum BDNF in speed group pre- and post-exercise on the first exercise (*p* = 0.159), pre- and post-exercise on the last exercise (*p* = 0.892). There was no significant increase in serum BDNF in inclination group on all parameters (*p* > 0.05). The serum BDNF is actually a neurotropic factor that affects not just the neuronal system, but also molecular energy and metabolism regulation. This serum is dependent on the aerobic capacity, lactate production, muscle calcium uptake, and muscle fiber type used in exercises. Furthermore, the serum BDNF is increased by treadmill exercises in escalated speed.

**Conclusion**: Treadmill exercises with average speed escalation increase the serum BDNF.

## Introduction

The fall rate among adults 20–45 years old has reached 18% of the population and has gradually increased proportionally with age and has risen to 35% on 65 years old and above (Talbot et al., 2005). Balance disorder is one of the causes of falling in human life. Balance is influenced by the neurological system: somatosensory, sensorimotor integration, motoric planning; also, by musculoskeletal system: verticality, postural and movement control. The contextual system integrates them all such as, environment, support surface, lighting, gravity, and physical characteristic (Kloos and Heiss, 2007). One Leg Stance (OLS) test is a simple static balance measurement that is easily applicable with minimal equipment. Springer et al. (2007) showed the OLS in a healthy population of various groups of age, OLS abnormality relates to frailty, independency of activity daily living, and high risk of fall status (Boer et al., 2014).

Our brief observation of OLS test in Rehabilitation Medicine Outpatient Clinic Dr. Soetomo General Academic Hospital on healthy people of 26–54 years showed 78% of 57 people had less than 50 s of OLS with their eyes closed. This showed a phenomenon of balance disturbance in a young and productive age that might contribute to the 18% fall in numbers as outlined above. Treadmill exercise is an aerobic training that is used to improve gait function; it also has two components (inclination and speed). Steib et al. (2017) showed perturbation using treadmill to stimulate specific adaptations in dynamic balance control during ambulation. The treadmill creates a constant challenge of the postural control system during walking (Steib et al., 2017). Pirouzi et al. (2014) showed that after a treadmill exercise, there is a significant increase in the berg balance score of the healthy older population. Shimada et al. (2004) revealed that there was an increased value of functional reach test and OLS value in older adult population after a bilateral separated treadmill exercise. This also showed an improvement in stability and mobility in an upright position. However, little evidence was found about OLS test in healthy young adults and there are no clear comparisons between inclination and speed treadmill as a suggested exercise.

Brain Derived Neurotropic Factor (BDNF) is essential for neuronal system, metabolism, peripheral and central homeostatic environment such as, regulating glucose oxidation, reduces blood glucose and increases insulin sensitivity (Knaepen et al., 2010). BDNF are neurotrophines expressed in brain, muscles, spinal cords, peripheral nerves (Binder and Scharfman, 2004). One of the mechanisms of balance function is manifested by the BDNF (Seo et al., 2010). It is also a superior facilitator for excitability, neuronal defense and differentiation also synaptic transmission through B tyrosine kinase receptor (TrkB; Binder and Scharfman, 2004; Rasmussen et al., 2009; Maqsood and Stone, 2016). BDNF affects the axonal terminal and dendrite growth, which are essential for memory and learning system. After 5 days of treadmill exercise in a rat, the BDNF was increased and expressed in its muscles (Gómez-Pinilla et al., 2002; Seo et al., 2010). In human trial, BDNF were increased after balance exercise using a treadmill (Zoladz et al., 2014). Peripheral BDNF can be measured through blood serum or plasma, considering its ability to pass blood brain barrier (Piepmeier and Etnier, 2014). Central BDNF is induced by PGC-1α and FNDC5 expressed after acute exercise as the escalation of physical activity and soon after central BDNF was released, they passed blood brain barrier and circulated peripherally. BDNF were suggested to decrease the neuronal degeneration after physical exercise (Gómez-Pinilla et al., 2002; Wrann et al., 2013). Sleiman et al. (2016) reported BDNF expression through D-β-hydroxybutyrate (DBHB) originated in the liver, DBHB then inhibited histone deacetylase (HDAC) 1, 2, 3 which first inhibited BDNF. Through this mechanism, DBHB induced the expression of BDNF.

The aim of this study is to compare BDNF value before and after moderate-intensity treadmill with increased speed and increased inclination in untrained young healthy adult males. We hypothesized that there would be a significant rise of serum BDNF as an immediate and adaptive response after the moderate-intensity treadmill exercise with a gradually increased inclination and speed, and also there were significant differences in BDNF value between two groups.

## Materials and Methods

### Study Design and Inclusion Criteria

The design of this study was a randomized pre- and post-test group. Twenty young healthy males were recruited in the study. The subjects were divided into two groups: inclination group (mean age 31.3 ± 3.04 years) and speed group (32.3 ± 2.31 years). Inclusion criteria were healthy adults according to WHO guidelines age within 26–37 years old with body mass index (BMI) 18–22.9 kg/m^2^ and signed the informed consent form. Participants were excluded if they had routine aerobic exercise at least two times per week, previous history of ischemic heart disease, restrictive/obstructive respiratory tract disease, and neuromusculoskeletal disease, systole blood pressure exceed 120 mmHg and diastole less than 60 mmHg, had vestibular and proprioceptive disturbance, and range of motion of both ankles for plantar flexion <45° and dorsiflexion <30°. This study was approved by ethical committee of Dr. Soetomo General Academic Hospital Surabaya Indonesia with ethical clearance no. 0206/KEPK/IV/2018.

### Protocols

Eligible subjects were asked to stand barefoot on the right limb and then left limb, with the other limb raised so that the raised foot was near but not touching the ankle of their stance limb. The subject was also asked to cross his arms over the chest and close their eyes. The investigator used a stopwatch to measure the amount of time the subject was able to stand on one limb. Time commenced when the subject raised their foot off the floor and ended when he used his arms, used the raised foot (moved it toward or away the standing limb or touched the floor), moved the weight-bearing foot to maintain his balance (rotated foot on the ground), a maximum of 50 s had elapsed. Time will be recorded.

**Figure 1:**
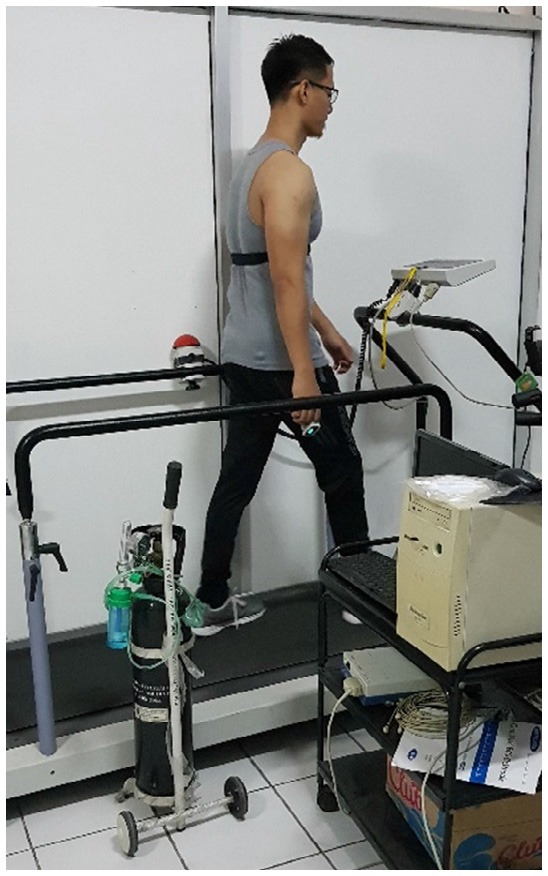
Inclination group participants using Polar H10 (Figure permissions had been approved by subject).

Treadmill EN-Mill^®^ 2007 were used as walking exercise devices. We utilized Polar H10 heartbeat sensors for the heart rate, installed to the participant’s chest and connected through blue-tooth to smart phones (as shown in Figure 1). The study was divided into two intervention groups, the inclination group using the Balke protocol (Froelicher et al., 1975) and the speed group, using athlete led protocols (Hamlin et al., 2012). The study was about 30 min long. A 5-min warm-up was initiated before the exercise then we had core exercises which lasted for 20 min and a 5-min cool-down for straight 2 weeks with a frequency of three times each week.

**Figure 2:**
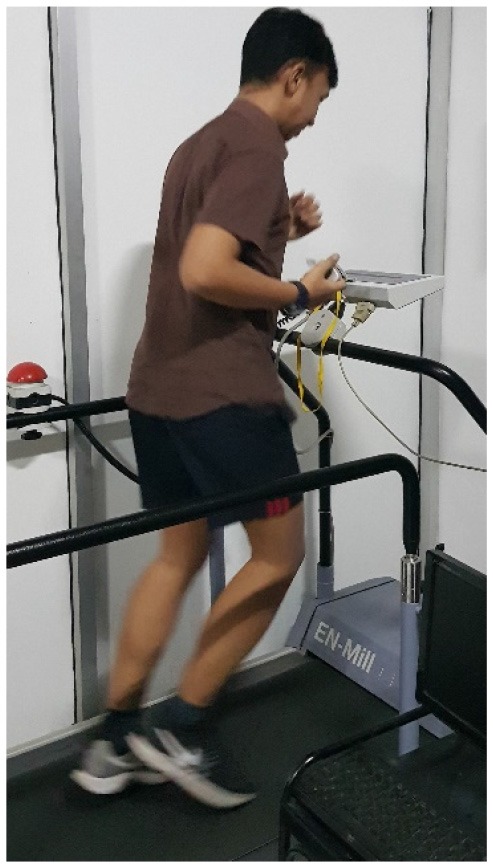
Speed group participants using Polar H10 (Figure permissions had been approved by subject).

Blood samples were taken from all groups 30 min before the exercise, and 30 min after the exercise on the first and the last training program. Blood drawn and put in plain tube (without any activator) and kept in −80°C refrigerator. BDNF were measured using IBL Human BDNF ELISA kit Catalog no. BE69099 and Lot No. E30/2018J. The inclination groups were subjected to 3 km/h speed with gradual increase of the inclination starting from 2.5%, 5%, 7.5%, 10%, 12.5%, 15%, 17.5%, 20%, 22%. The inclination was increased every 1 min until the participant reached the target heart rate (70% maximum heart rate). The heart rates were recorded every minute, the inclination was raised. Speed groups started with 5 km/h and added 1 km/h every minute with 0% inclination until the participant reached the target heart rate (70% maximum heart rate; as shown in Figure 2). Serum BDNF was measured using IBL ELISA Human BDNF kit. Assay description: 90 min incubation (37°C) + 60 min (37°C) + 30 min (37°C) + 30 min (37°C) = 3 h, 30-min total incubation time. Sample volume 100 μL proper diluted human serum. Standard range: 0/31.2–2,000.0 pg/mL. Sensitivity <2 pg/mL (Anonym, in press).

### Data Analysis

We observe the average value of BDNF, delta BDNF which show the difference of BDNF value pre- and post-exercise in each group. For statistic measurement, we used Kolmogorov–Smirnov for homogeneity test. The results were normal distribution, therefore authors used independent samples test to measure significance of each group and paired *T*-test to measure significance between two groups using IBM SPSS statistic 23 system.

## Results

This study was conducted for 2 weeks and included 20 young healthy males, and then all the participants were divided into two groups by randomized ballot. The participants were employees and residents medical doctors of Physical Medicine and Rehabilitation in Dr. Soetomo General Academic Hospital, Surabaya. Ten young men were grouped to utilize the inclination and 10 men for the speed. We presented the demographic data with normality test using independent samples test and exhibited normal distribution in both groups with *p* > 0.05 (Tables 1 and 2).

**Table 1:** Demographic characteristic.

	Groups	*N*	Mean	SD	*p*
Age (years)	Inclination	10	31.33	±3.04	0.443
	Speed	10	32.30	±2.31
Body weight (kg)	Inclination	10	64.11	±6.62	0.843
	Speed	10	64.80	±8.15
Height (cm)	Inclination	10	169.22	±7.21	0.876
	Speed	10	168.70	±7.18
BMI (kg/m^2^)	Inclination	10	22.20	±1.14	0.492
	Speed	10	22.64	±1.54	

*Significant if p < 0.05 using independent samples test*.

**Table 2:** Normality test of CECAOLS in both groups before training.

	Groups	*N*	Mean	SD	*p*
Closed eyes, crossed arms,	Inclination	10	25.39	±12.11	0.338
Left, pre (s)	Speed	10	34.44	±25.78
Closed eyes, crossed arms,	Inclination	10	18.97	±15.98	0.555
Right, pre (s)	Speed	10	24.51	±23.02	

*Significant if p < 0.05 using independent samples test*.

### Inclination Group

**Figure 3:**
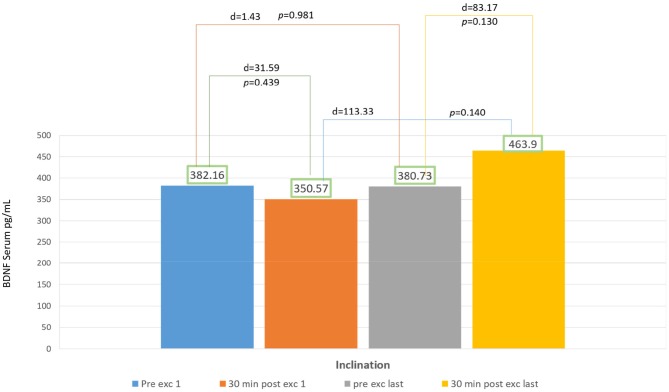
Brain derived neurotropic factor (BDNF) serum result in inclination group.

In 2 weeks of exercise, the inclination group exhibited an escalating trend of BDNF serum, they exhibited an acute reduction of BDNF serum on the first day and only 18% escalation of BDNF serum after acute exercise on the final day. There was no significant increase of delta BDNF serum value in between the session from the first to the last day, and also the pre- and post-exercises from the first to the final day, with *p* > 0.05 (Figure 3).

### Speed Group

**Figure 4:**
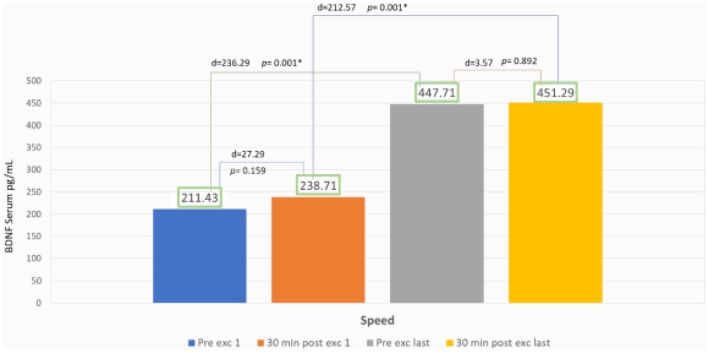
BDNF serum result in speed group.

The speed group exhibited a significant increase of BDNF serum with 111% escalation average. Comparing the BDNF serum of the pre-exercises from the first to the last day with *p* = 0.001 (*p* < 0.05), and also comparing 89% escalation average of BDNF from the first exercise to the last with *p* = 0.001 (*p* < 0.05) but there was no significant increase of average BDNF serum between session exercises compared with post-exercise on the first day with *p* = 0.159 (*p* > 0.05) and also comparing the first day of pre- and post-exercises with *p* = 0.892 (*p* > 0.05; Figure 4).

### Inclination vs. Speed Group

**Figure 5:**
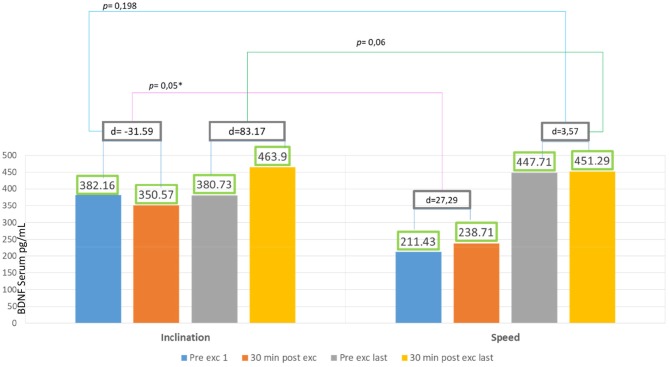
The difference of BDNF serum result in speed and inclination group.

There was significant escalation found in difference of pre-post exercie day 1 in inclination group compared to difference of pre-post exercise day 1 in speed group (*p* = 0.05). But there was no significant value found in difference of pre-post exercise last day in inclination group compared to difference of pre-post exercise last day in speed group (*p* = 0.06; Figure 5).

### Correlation of BDNF to Closed Eyes Crossed Arms One Leg Stance

In our study, we found there were significant negative correlations between CECAOLS before exercise with BDNF changes of the first day in speed group (*p* = 0.028). Also, there were negative significant correlations between CECAOLS after the last exercise with BDNF changes in last day of inclination group (*p* = 0.035; Table 3).

**Table 3:** Correlation of CECAOLS and BDNF changes in both groups before and after training.

		Speed		Inclination	
		Δ BDNF	Δ BDNF	Δ BDNF	Δ BDNF
		exercise 1st day	exercise last day	exercise 1st day	exercise last day
CECAOLS Pre-Exercise	D	*r* = −0.688	*r* = 0.241	*r* = 0.203	*r* = −0.315
		*p* = 0.028*	*p* = 0.502	*p* = 0.600	*p* = 0.409
	S	*r* = −0.433	*r* = 0.168	*r* = 0.232	*r* = 0.278
		*p* = 0.212	*p* = 0.643	*p* = 0.548	*p* = 0.469
CECAOLS Post-Exercise	D	*r* = 0.081	*r* = −0.239	*r* = 0.311	*r* = −0.701
		*p* = 0.824	*p* = 0.506	*p* = 0.415	*p* = 0.035*
	S	*r* = −0.129	*r* = −0.036	*r* = 0.332	*r* = −0.467
		*p* = 0.723	*p* = 0.921	*p* = 0.383	*p* = 0.205

*Significant if p-value <0.05 with Pearson Correlation Test.

## Discussion

### Demographic Characteristic

Normal distribution of the demographic characteristics shows homogeneity of ages, weight, height, as well as BMI also showed the average CECAOLS value of participants in both groups using the independent sample test (*p* > 0.05; Table 1). This showed that the parameters had an average variety. The normal distribution of test in various characteristics ranging from the first and last days of pre- to post-exercises with *p* > 0.05 (Table 2). It also showed that both groups had no significant value (*p* > 0.05). Our subjects were 20 young healthy males because we wanted to eliminate the hormonal influence on BDNF production. Scharfman and MacLusky (2006) described that estrogen and BDNF may share common signal transduction pathway, effectors, and may interact to exert their effects. The possibility was BDNF synthesis is induced by estrogen. Sohrabji et al. (1995) reported that estrogen rapidly up-regulates BDNF mRNA in rats.

### Role of BDNF in Inclination Group

According to Karp, depending on the eccentricity and velocity of contracted muscles, some movement directly recruits fast-twitch muscle fiber. The recruitment of fast-twitch muscles depends on exercise intensity, variety, and goals (Karp, 2001). Gradual inclination requires more muscle compared to speed treadmill at the ground level. Muscles recruited also works to maintain balance while inclined (Salim et al., 2012). In our study, the graphic representation for inclination group showed a decrease in BDNF serum value after 30 min of exercising on the first day (Figure 3). This phenomenon might occur due to the changes in muscle fiber recruitment occurring in treadmill exercises with gradual inclinations. First sequence in a particular amount of time showed individuals recruited fast-twitch muscle fiber, this caused the escalation of BDNF serum (Figure 3). Fast-twitch muscle produces higher and stronger forces of contraction with peak time of 40–60 ms compared to slow-twitch muscle fiber (Maglischo, 2015).

In our study, within a particular time, there were changes of muscles recruitment from fast to slow-twitch muscle fiber that might cause the reduction of BDNF serum in 30 min after exercise in the first day. Walking inclined to the ground level forces the center of mass to displace the forward foot center, and at the same time, the gravity accelerated body to move forward. Momentum is also generated by trunk displacement of both legs (Leroux et al., 2002). Aerobic capacity is affected by mitochondria regulation-dependent Ca^++^ that increases lactate concentration (Stallknecht et al., 1998).

BDNF are induced by many elements in body such as lactate, amount of Ca^++^, aerobic capacity, and also muscle fiber type used in exercising. Schiffer et al. (2011) reported the increase of BDNF serum as a response to lactate infusion in resting states.

Calcium intracellular activated CAMP Response Element Binding (CREB) will induce BDNF (West et al., 2001). There is production of BDNF in organs such as neurons, brains, muscles, and thrombocytes (Klein et al., 2011). Sakuma and Yamaguchi (2011) reported that muscle fiber also acted in neurotrophin production, there was still controversy that type I muscle fiber produces more neurotrophin than type II, but it shows in their studies that type II produce more BDNF than type I. It is possible that the escalation trends of BDNF serum in inclination group might be caused by lactate and intracellular calcium, although it is not statistically significant. For further study, longer duration and frequency might be necessary for exercise using inclination treadmill to produce BDNF serum (Figure 3) also its relation with lactate and intracellular calcium.

### Role of BDNF in Speed Group

In the speed group, there was no significant increase of BDNF serum intra-session from the first to the last day (Figure 4). We found this information to correlate with Gold et al. (2003) and Ferreira et al. (2011) that reported that there was no significant increase of BDNF serum after 30 min static cycle and treadmill exercise in rats. Our findings were contrary with Rojas Vega et al. (2006) and Zoladz et al. (2008) that showed a significant increase of BDNF serum after 30 min static cycle in athlete subject and after 40 min of static cycle with moderate intensity in young adult male subject.

Ferris et al. (2007) explained that BDNF production were dependent of diurnal factors and exercise duration. We thought that longer duration of intra-session exercise might improve BDNF serum value (Figure 4). In speed treadmill exercises, fast-twitch muscle fiber will be recruited, and then adapted within a period of time to the slow-twitch muscles. It may be related to more nerve responses which will increase the cell response to produce BDNF peripherally (Figure 5). Lieberman et al. (2001) reported that high-intensity interval exercises increase the type I muscle proportion to type II muscle fibers. These conditions cause glycogenolysis and more lactate production that in turn affects the serum BDNF. Baseline BDNF increases before exercise significantly in the first day compared to the last day. In our study, the escalation reaches about 111%, this phenomenon showed chronic adaptation of tissues involved and affected by BDNF, it might also relate to tissue self-repair capacity that regulates metabolism and energy during weeks of exercise. It is suitable with who revealed BDNF peripheral capacity in metabotropic and neuroendocrine. We also found significant increase of BDNF serum comparing the first and last day of exercise. The escalation reaches about 89%, showed responsive adaptive capacity in acute exercise for over 30 min. In speed exercises, there was repeated reactivation of fast-twitch muscle fiber after the use of slow-twitch muscles, showing the change of the sequence of muscle fiber recruitment. Maglischo reported that fast-twitch muscle fiber had bigger diameter of motoric nerve fiber that is consequently recruited if one’s body needed to be near maximal power and forces to accomplish exercises (Maglischo, 2015).

### Serum BDNF in Inclination and Speed Groups

Figure 5 showed significant difference in delta inclination and delta speed intra-session exercise first day and intra-session last day. This showed treadmill exercise with speed increasing is better in producing BDNF. In inclination group, the baseline BDNF serum is higher 55% than speed group. It might be affected by physical activity, as Currie et al. (2009) reported that habitual physical activity and aerobic capacity affect the serum BDNF value to active male and female human subject aged between 18 and 57 years. Rasmussen et al. (2009) also reported that there were significant BDNF production during prolonged exercise, and that exercise might be a major source for increased plasma BDNF during exercise in healthy men subjects aged between 22 and 40 years old.

### Correlation of BDNF to Closed Eyes Crossed Arms One Leg Stance

At the first exercise in speed group, the low value of CECAOLS before exercise caused body compensation by increasing nerve activation which reflected by the BDNF value. In inclination group, there was positive correlation between CECAOLS and BDNF. The low value of CECAOLS led to small increasing of BDNF value, it was not significant. OLS was one of the balance measurement, treadmill exercise could be one of the balance exercise (Shimada et al., 2004). Seo et al. (2010) reported that BDNF was increased after balance training.

At the last exercise in inclination group, there were negative correlation between BDNF and CECAOLS. That is, low value of BDNF led to significant increase of CECAOLS of the right leg. In the speed group, there was also negative correlation between BDNF and CECAOLS. Low value of BDNF led to small changes of CECAOLS of the right leg. This showed inclination exercise will increase BDNF more efficient to achieve higher CECAOLS. McGeown et al. (2016) reported that there was positive relationship between BDNF and aerobic exercise which reflected by significant improvement of cognitive function, recovery of balance function such as single-leg firm stance, average velocity of center of pressure (COP), area of COP, and tandem firm stance in healthy young individuals.

In speed group, there was negative correlation between BDNF and CECAOLS right leg pre-exercise that becomes positive correlation after exercise in the first day (Trend Trend Graphs 1 and 2). After 2 weeks of serial exercise, it showed positive correlation then become negative correlation, possibly due to adaptation mechanism (Trend Trend Graphs 3 and 4). This might be caused by the faster adaptation of aerobic capacity and muscle fiber recruitment that affected the BDNF production, as we have stated above.

**Trend Graph 1:**
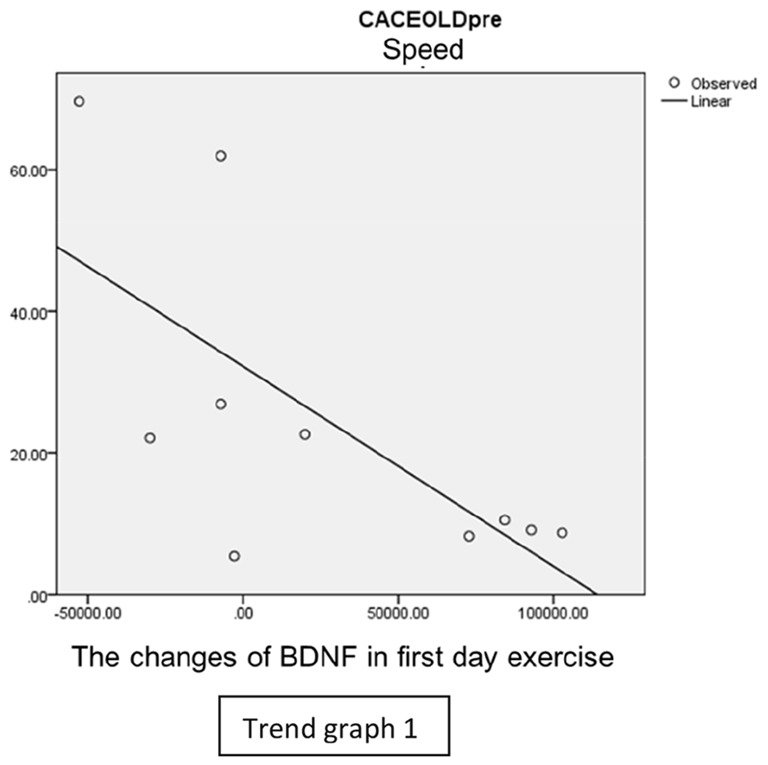
Correlation of BDNF and CECAOL Dextra in Speed Group Pre Exercise First Day.

**Trend Graph 2:**
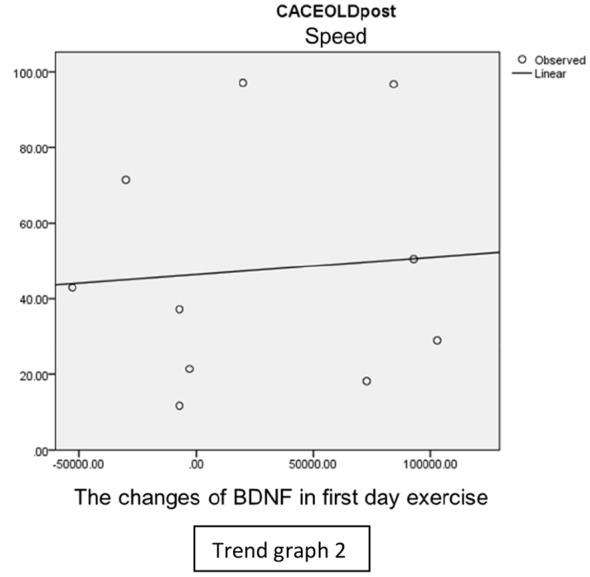
Correlation of BDNF and CECAOL Dextra in Speed Group Post Exercise First Day.

**Trend Graph 3:**
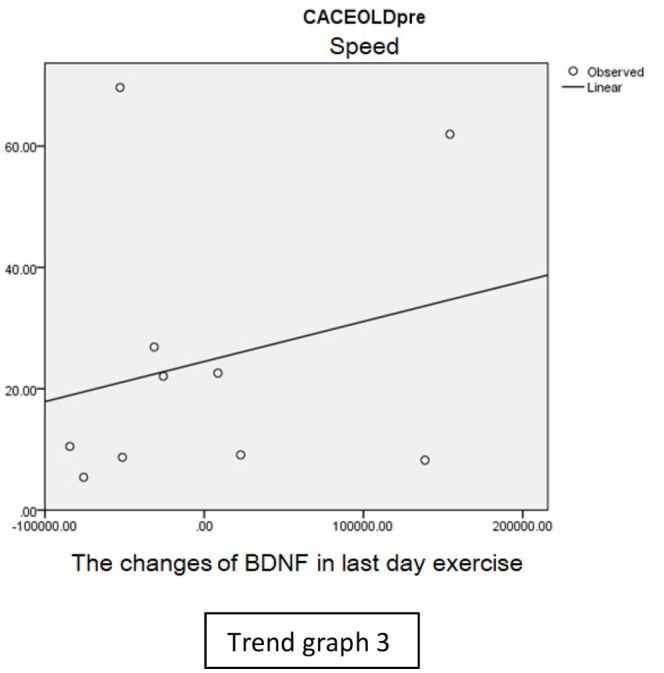
Correlation of BDNF and CECAOL Dextra in Speed Group Pre Exercise Last Day.

**Trend Graph 4:**
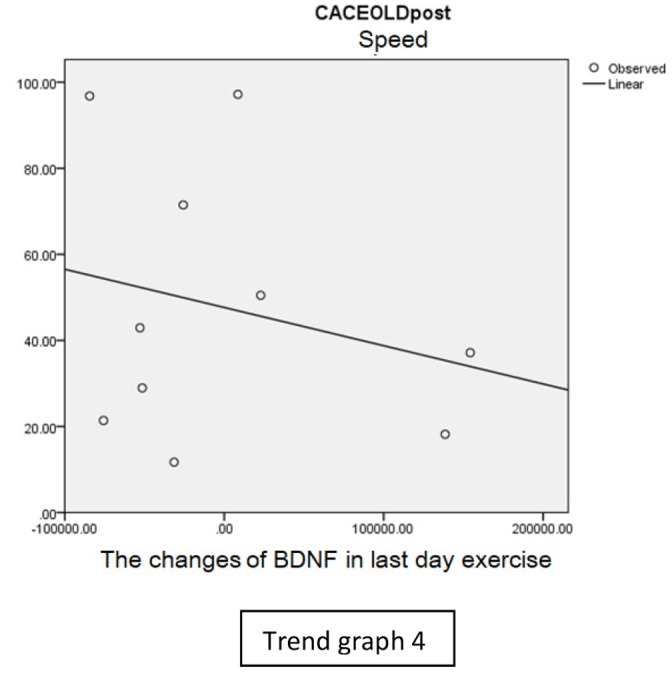
Correlation of BDNF and CECAOL Dextra in Speed Group Post Exercise Last Day.

In the inclination group, there was positive correlation between BDNF and CECAOLS right leg pre-exercise that become even more stronger positive correlation after exercise in the first day (Trend Trend Graphs 5 and 6). After 2 weeks of serial exercise, it showed negative correlation then become stronger negative correlation (Trend Trend Graphs 7 and 8). This might be caused by prolonged adaptation of aerobic capacity and muscle fiber recruitment that affected the BDNF production, as we have stated above.

**Trend Graph 5:**
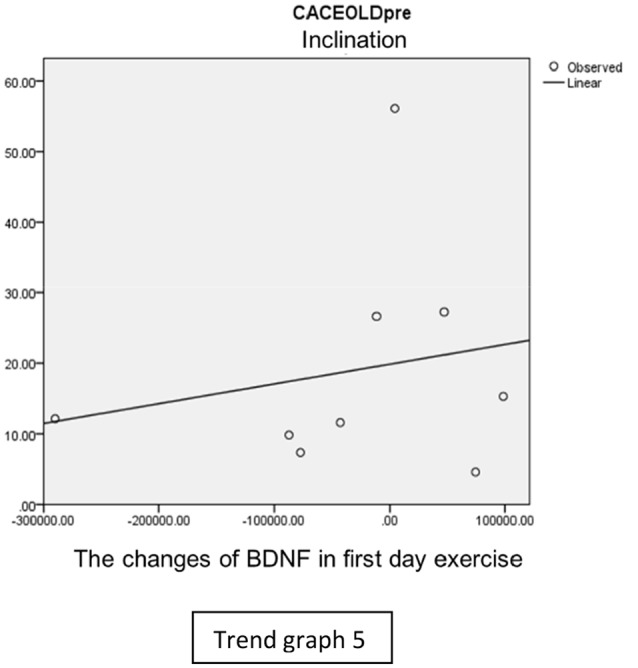
Correlation of BDNF and CECAOL Dextra in Inclination Group Pre Exercise First Day.

**Trend Graph 6:**
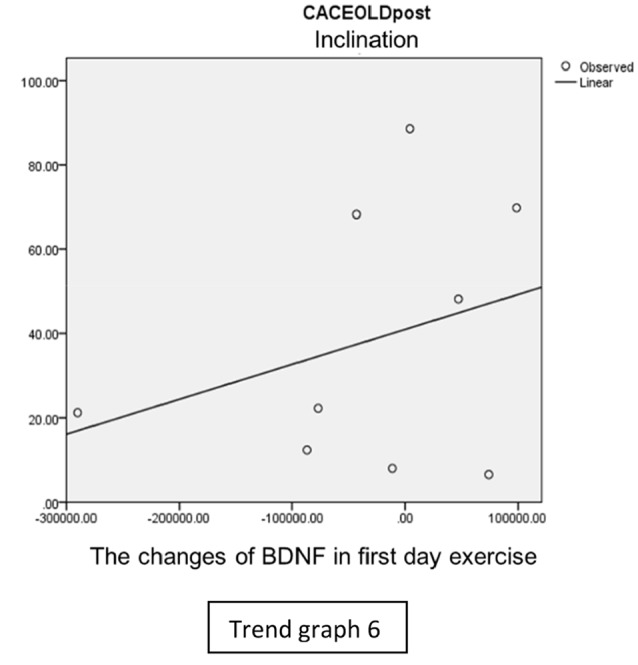
Correlation of BDNF and CECAOL Dextra in Inclination Group Post Exercise First Day.

**Trend Graph 7:**
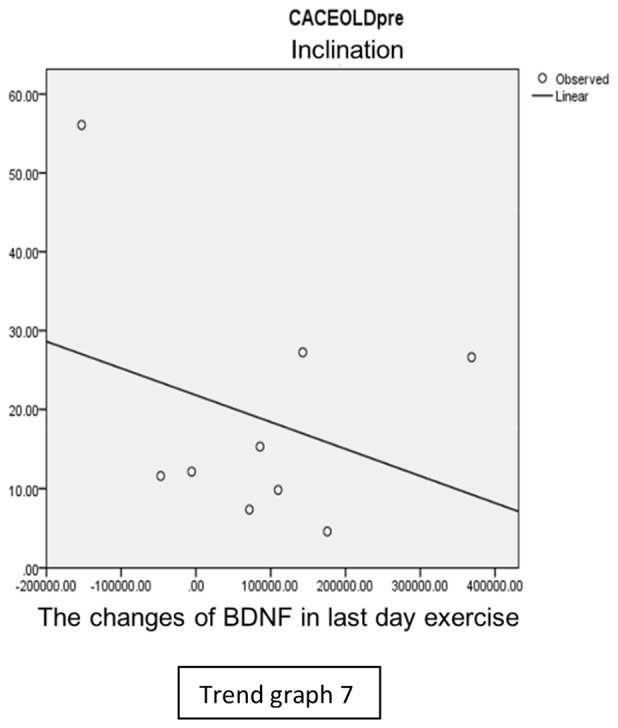
Correlation of BDNF and CECAOL Dextra in Inclination Group Pre Exercise Last Day.

**Trend Graph 8:**
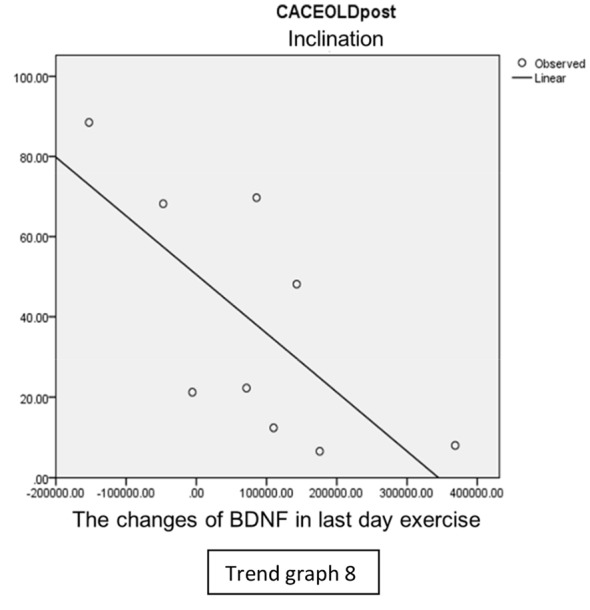
Correlation of BDNF and CECAOL Dextra in Inclination Group Post Exercise Last Day.

## Study Limitation

Specific daily physical activities were not observed and might affect the BDNF serum objectively, it could be one of our confounding factors. Generalization of exercise is limited only in age characteristic, healthy adult male as hormonal factor can be a confounding factor. Therefore, we suggest further study in female subjects. The 30 min duration and 2 weeks intervention period of exercise did not show any significant escalation of BDNF serum in inclination group. For future study, longer duration and intervention period might be ideal to increase BDNF serum significantly using inclination treadmill.

## Conclusion

There were significant increases in BDNF serum before and after 2 weeks moderate-intensity treadmill exercise with gradual speed escalation, however there was no significant increase of BDNF serum before and after treadmill exercise with gradual inclination escalation, and also there was significant difference of BDNF value between inclination and speed group for 2 weeks training in young healthy adult male with occult balance disturbance.

In the future, we suggest further study of occult balance disturbance. The treadmill exercise particularly with gradual speed increased are beneficial in developing BDNF serum with frequency three times per week in 2 weeks straight to young adult healthy male with occult balance disturbance. Also, the combination of speed and inclination treadmill exercise might enhance the serum BDNF.

## Ethics Statement

This study was carried out in the accordance with recommendations of Balke and Athlete led protocols, Froelicher et al. (1975) and Hamlin et al. (2012) with written informed consent from all subjects. All subjects gave written informed consent in accordance with Declaration of Helsinki. The Protocols were offered approved by Elizeus Hanandito, Anestesiologist-consultant, Chief of Ethical Commitee Dr. Soetomo Hospital Surabaya Indonesia.

## Author Contributions

All authors listed have made a substantial, direct and intellectual contribution to the work, and approved it for publication.

## Conflict of Interest Statement

The authors declare that the research was conducted in the absence of any commercial or financial relationships that could be construed as a potential conflict of interest.
